# Deep learning-based school attendance prediction for autistic students

**DOI:** 10.1038/s41598-022-05258-z

**Published:** 2022-01-26

**Authors:** Mohammed Jarbou, Daehan Won, Jennifer Gillis-Mattson, Raymond Romanczyk

**Affiliations:** 1 Department of Systems Science and Industrial Engineering, Binghamton University-State University of New York, Binghamton, NY 13902 USA; 2 Department of Psychology, Binghamton University-State University of New York, Binghamton, NY 13902 USA

**Keywords:** Engineering, Human behaviour, Psychology and behaviour

## Abstract

Autism Spectrum Disorder is a neurodevelopmental disorder characterized by deficits in social communication and interaction as well as the presence of repetitive, restricted patterns of behavior, interests, or activities. Many autistic students experience difficulty with daily functioning at school and home. Given these difficulties, regular school attendance is a primary source for autistic students to receive an appropriate range of needed educational and therapeutic interventions. Moreover, school absenteeism (SA) is associated with negative consequences such as school drop-out. Therefore, early SA prediction would help school districts to intervene properly to ameliorate this issue. Due to its heterogeneity, autistic students show within-group differences concerning their SA. A comprehensive statistical analysis performed by the authors shows that the individual and demographic characteristics of the targeted population are not predictive factors of SA. So, we used the students’ recent previous attendance to predict their future attendance. We introduce a deep learning-based framework for predicting short-and long-term SA of autistic students using the Long Short-Term Memory (LSTM) and Multilayer Perceptron (MLP) algorithms. The adopted algorithms outperform other machine learning algorithms. In detail, LSTM increased the accuracy and recall of short-term SA prediction by 20% and 13%, while the same scores of long-term SA prediction increased by 5% using MLP.

## Introduction

Autism Spectrum Disorder (ASD) is a neurodevelopmental disorder characterized by deficits in social communication and interaction as well as the presence of repetitive, restricted patterns of behavior, interests, or activities^1,2^. Although its risk factors are yet not fully defined, different genetic, biological, and environmental factors are addressed in the literature as contributing to the development of ASD^2,3^. Approximately 1.5–2.0% of the children in the US are diagnosed with ASD.

Many autistic children experience difficulties with a range of areas of daily functioning at school and home, making it paramount that they have access to receive interventions and learning opportunities, especially those offered at school^4,5^. For example, attending public school enables autistic students to interact with their neurotypical or non-autistic peers, which may increase their social development, a key area of need for children with ASD. However, some autistic children attend special education schools or self-contained classrooms that provide intensive, specialized interventions^6^.

Recent reports suggest that autistic students miss school more than other clinical populations, leading to fewer opportunities for these students to benefit from school-based interventions^7^. For example, 5–28% of neurotypical or non-autistic students are reported to have missed school days while this percentage jumps to 40–53% among autistic students^7^. Similarly, the percentage of chronic absenteeism (CA; defined as missing more than 10% of the annual school days) among non-autistic students is 13% relative to 23% for autistic students^8,9^. These statistics clearly illustrate that SA disproportionally affects autistic children and can serve to negatively impact the effectiveness of ASD school-based interventions^6–8^.

At present, there is growing research on risk factors for SA in autistic children. Some areas that require further attention include (1) examination of gender, anxiety, depression, and challenging home settings; (2) examination of possible associations between SA and specific child characteristics^7,9^. For instance, students with particular health conditions (e.g., asthma) miss more school days when they experience severe symptoms^7^; and (3) group-level comparison between autistic and non-autistic students regarding SA risk factors. Besides the importance of these studies, the problem of SA prediction needs also be addressed considering the possibility of providing timely interventions to improve the SA. To the best of our knowledge, this is the first study introducing Machine Learning-(ML) and Deep Learning-(DL) based frameworks for predicting SA of autistic students. The following paragraphs discuss the challenges of the SA prediction problem and the advantages of using ML and DL techniques over the conventional statistical analysis tools.

SA prediction aims to establish the probability for each student of the number of missed school days in the future. If sufficiently accurate, such information could allow school districts and at-home caregivers to understand SA patterns and perhaps divert attention and resources to specific children. This would enable students to attend school regularly and benefit from school-based interventions and services.

**Figure 1:**
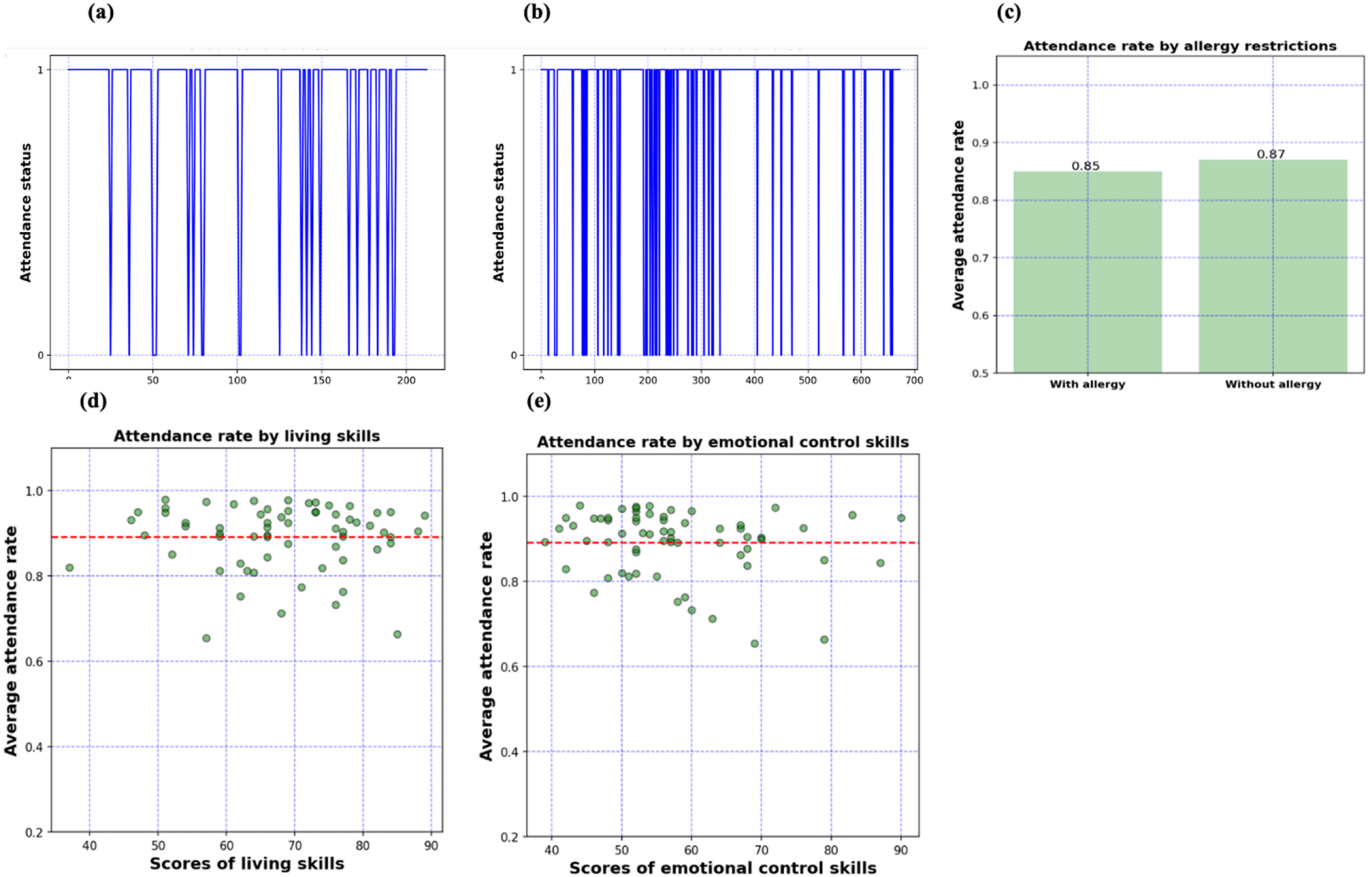
Heterogeneity and time-variant nature of SA behavior. (**a**) SA pattern for a student with an average attendance rate (85%). (**b**) Another pattern for a different student with a similar average rate (88%) but less frequency. (**c**) Attendance rate for students with/without allergy. (**d**) Attendance rate by the score of living skills. (**e**) Attendance rate by the score of living emotional control skills. The figures show no association between SA behavior and the individual characteristics of autistic students. The figures also show that SA behavior is better to be predicted/investigated at the individual level.

SA prediction is beneficial yet challenging. The challenges of SA prediction are associated with (1) the complexity of SA risk factors as being presented at multiple levels of different systems^10^ (2) the variety of ASD symptoms in terms of the type and severity^5^, and (3) the time complexity of SA behavior of the students themselves^11^. For instance, we studied the association of SA behavior of 120 autistic students with 14 different risk factors. The results in Fig. 1 show that students with equal or similar attendance rates show very different individual attendance patterns. The figure also shows that autistic students with different risk factors (e.g., food allergy) show a similar attendance rate.

Importantly, these findings support a hypothesis that the group-level analysis of SA risk factors does not necessarily explain the SA behavior of autistic students at the individual level. In other words, the challenges of SA prediction, supported by the findings of our statistical analysis, limit the viability of SA risk factors in predicting the individual SA behavior of autistic students in the future. To address this gap in the literature, the authors aim to explore and validate the viability of using the students' SA history to predict their future attendance.

The benefits of using SA history are manifold considering that it is: (1) more available and less expensive to be collected compared to other risk factors, (2) time-variant and captures the time complexity of SA behavior where other risk factors are static, (3) univariate which makes SA prediction less challenging than using multiple factors which are unequally associated with SA.

Predicting SA at the individual level requires mining the SA history of each student. The authors decided to recast the SA prediction problem into a time-series based sequence prediction. Therefore, we used the students' attendance and maladaptive behaviors, modeled as a time series, as input data to predict their SA and CA behaviors in the future. Methodology-wise, we used ML and DL techniques because they outperform the conventional methods (e.g., ARIMA). More details in this regard will be given in the following sections.

The main hypothesis of this research is twofold: (1) each autistic student shows different SA patterns (as shown in Fig. 1); and therefore, (2) SA is better predicted at the individual level. These hypotheses led the authors to utilize a framework that employs a combination of DL, ML, and time series modeling techniques to model and predict the individual SA and CA. These techniques are adopted because they outperform the conventional statistical techniques (e.g., ARIMA) in learning the complex patterns and long-range dependencies of the temporal data (e.g., SA behavior).

The results are expected to provide early predictions of SA and which students might be at risk of CA in the future. The present research uses a real dataset for a population of 120 autistic students. The data was collected at a private special education school in a mid-Atlantic state. More details about the data will be provided in the following sections.

The first objective of this research is to propose a short-term prediction framework to predict the SA at the individual level. This framework efficiently predicts whether a particular student will attend school over a prediction horizon of 10 school days. The second complementary objective is to propose a long-term prediction framework as to whether a particular student will be at risk of CA over the upcoming three months. CA needs to be predicted early enough because it is challenging and demands intensive and systematic interventions to be in place^8,9^.

The main contribution of this research is to introduce an ML/DL-based framework for short-term SA and long-term CA prediction. This objective has been set by the authors to address the following gaps in the literature:

1. Provide accurate predictions of SA and CA behavior using DL, ML, and time series modeling.
2. This is the first research that predicts SA and CA of autistic students at the individual level with consideration of the heterogeneity of ASD.

## Literature review

### ASD risk factors and phenotypes

ASD is a neurodevelopmental disorder with a wide range of symptoms and levels of severity mostly impacting areas of social communication as well as the presence of repetitive, restricted patterns of behavior, interests, or activities^2^. Currently its etiology and risk factors are still not well-defined despite the research efforts dedicated to this purpose^12–14^. The main common risk factors are genetic^12^, demographic^14^, environmental^12,13^, and family-related^14^. The association between ASD and other factors, such as parental, perinatal, prenatal, and neonatal, are also investigated and discussed in the literature^14^.

The advances in genetics research led to a growing interest in discovering what causes ASD from a genetic perspective. This question is still challenging, and its answer is arguable. While many studies show that autism traits are heritable, the responsible gene factor(s) is (are) not commonly defined^14^. Some research shows that different gene expressions cause different traits or symptoms of ASD. On the contrary, other studies concluded that different traits could be linked to the same underlying genetic expression^14^.

As a parallel research stream to ASD diagnosis, a significant amount of research investigates ASD-related symptom patterns, psychiatric disorders, and medical conditions. For example, autistic children are reported to have different facial expressions and sleeping patterns, such as bedtime resistance, night waking, sleep anxiety, and many others^15,16^. Food aversion (e.g., eating refusal), social anxiety, and aggressive behavior are all considered as phenotypes of ASD^17,18^.

**Figure 2:**
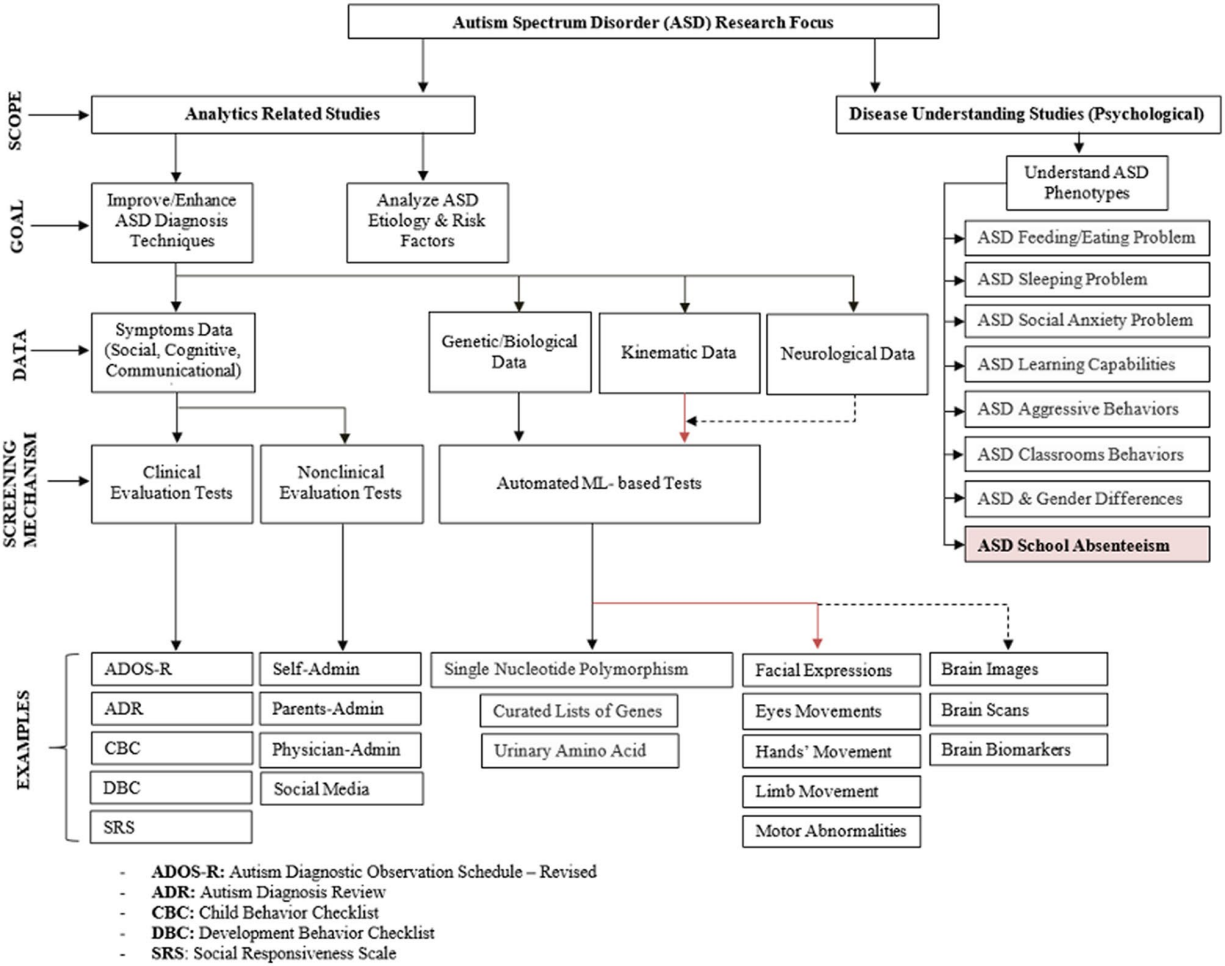
Schematic diagram of ASD literature review. This research focuses on SA as one of the ASD phenotypes.

Growing research explores the differences in the academic achievement of autistic students^19^. In this regard, autistic students show less participation, poorer academic outcomes, and more consistent absenteeism compared to their neurotypical peers^7,8,10^. Other studies show that reading comprehension skills and educational engagement are also worse for autistic students^20^. In addition, autistic students have different social engagement and school-related behaviors compared to neurotypical students^7,20^. The predictors of these academic challenges are studied in the literature without being identified as autism-specific^11,20^. For example, autistic students show more problematic SA that might lead to academic underachievement without being definitely autism-specific^11^. Figure 2 layouts the literature on ASD.

### SA and CA risk factors

SA is problematic for its long-term impact on the students’ academic outcomes^6^. Recent reports show that 13–16% of US students are chronically absent^8,9^. This percentage represented eight million students in 2015^8,9^. The percentage of CA among autistic students is twice that of non-autistic students^9^. Given these alarming figures, the association between SA and ASD has been inadequately studied^10^. Challenges highlighted in this regard are manifold: (1) autistic students show different SA patterns, frequency, duration, and expression; (2) SA behavior appears to be idiosyncratic. Therefore, the population-based investigation does not necessarily represent an individual’s SA, and (3) SA is a time-variant due to the vulnerability of the autistic students to the surrounding environment^7^.

A significant amount of research focuses on studying the types of school absenteeism problems which are school refusal, truancy, school withdrawal, and school exclusion^10,11^. However, the relationship between SA types and their associated factors is yet to be well investigated^11^. Recently, an inclusive framework has been proposed to guide understanding the SA risk factors considering (1) the degree of association between the risk factors and the type of SA, and (2) students with and without disabilities^11^. In this research, we focus on missing a full-day type of absence among autistic students.

In the literature of typically developing students, the risk factors of SA are either individual, familial, or environmental^4,6^. While anxiety and poor social relationships are examples of individual risk factors^6,9^, familial risk factors include parental support and home atmosphere^10^. Individual risk factors also include many demographic factors such as age, gender, and the characteristics of the household. For example, students living in two employed parents show less SA^10^. In the same context, other risk factors studied in the literature include bullying^21^, alcohol consumption^22^, and household exposure factors^23^.

The school environment has a significant effect in this regard. Also, the transition between classes, grades, developmental stages, as well as learning demands is other challenging risk factors for SA^19^. The type of school is also shown to contribute to the absence rate of autistic students such that older students in mainstream schools show more SA^10,11^.

Awareness has been raised to the schools' role in managing and controlling SA through early and well-designed interventions^19^. SA prediction is critical for schools to effectively improve their students' attendance. To accomplish this, schools need to know, in advance, when and for how long each student might be absent. This will give the schools enough time to plan for proper and effective interventions.

### Statistical models for SA and CA prediction

Many research studies investigate the SA and school refusal factors using different statistical techniques. For example, the chi-square test and logistic regression have been used to analyze and compare the SA characteristics in autistic and non-autistic students^21^. Multivariate logistic regression model has been fit to investigate the association between multiple individual characteristics of autistic students and school refusal^21^. Statistical analysis is also used to explore the association between anxiety, social phobia, and SA among autistic students^24^. For typically developing students, different statistical analysis techniques are also used to test the significance of different risk factors as alcohol consumption^22^, asthma^23^, household food insecurity^25^. In the same regard, a meta-analytic review has recently shown the statistical significance of multiple risk factors^26^ of SA.

### Machine learning in education

ML is a set of powerful techniques widely used to analyze and obtain useful insights from multivariate and complex data. Interest is growing to harness ML capabilities in the area of education research. For typically developing students, the association mining algorithm is used to discover the students' behavioral factors that affect their e-learning courses^27^. Clustering algorithms are also used to assign students into homogeneous groups of similar learning styles^27^. Also, the students’ drop-out possibility is predicted using logistic regression and decision tree algorithms^27,28^. A neural network classifier is also used for predicting students’ outcomes^29^. Multiple ML models have been applied to predict the absenteeism of public school teachers^30^. Other recent research works focus on leveraging ML algorithms to predict students’ academic performance^31^. Few research efforts have been dedicated to developing a systematic review of ML applications in the education domain^32,33^.

SA is another research focus of education research literature. Intensive research work has been directed at defining the risk factors of SA^7,10,11,26^. To the best of our knowledge, ML and DL algorithms have not been used to predict the SA behavior of autistic students or any other child population. This research aims to fill this literature gap by introducing an ML/DL framework for SA and CA prediction among autistic students. In addition, it is important to mention that developing a new prediction algorithm for SA prediction is out of our scope in this research. Instead, we aim at adding to the literature by highlighting and validating the viability of ML/DL in algorithms in handling SA and the maladaptive behavior of autistic students.

## Results and discussion

### Short term SA prediction (univariate and multivariate)

**Figure 3:**
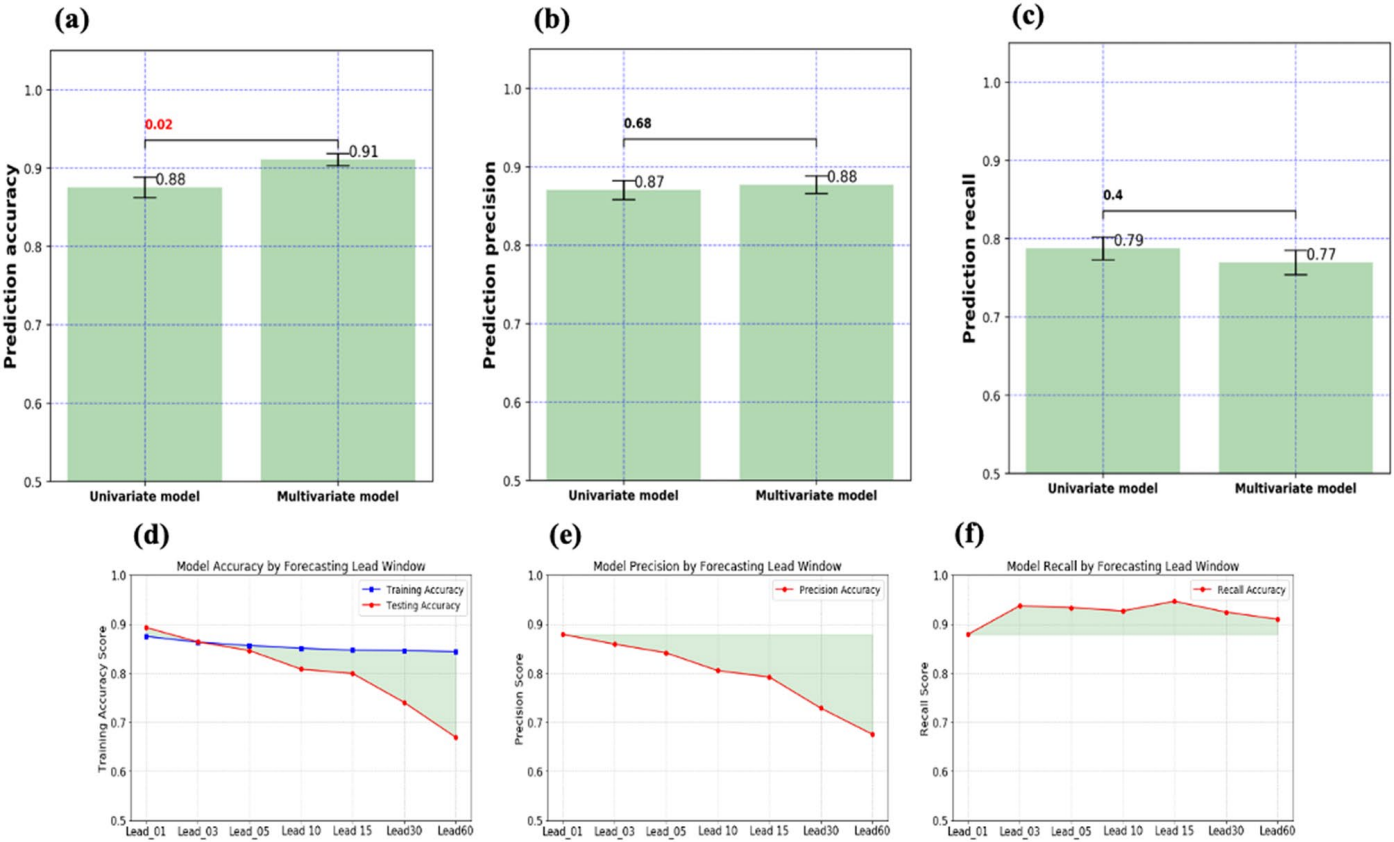
SA prediction performance of the proposed models. (**a**) Multivariate model outperforms the univariate model in terms of prediction accuracy (one-way ANOVA, $n$ = 125, $p$ = 0.02), (**b**) Prediction precision of both models is statically indifferent (one-way ANOVA, $n$ = 125, $p$ = 0.68). (**c**) Prediction recall of both models is statically indifferent (one-way ANOVA, $n$ = 125, $p$ = 0.40). Adding maladaptive behavior through multivariate model significantly improves the quality of SA prediction in terms of accuracy ($p=0.02\le 0.05)$. (**e**) Prediction accuracy changes by the lead value (days). (**e**) Prediction precision decrease by lead value (days). (**f**) Prediction recall change by lead value (days). The model is reliable to be used for predicting ten school days ahead with 90% accuracy and 80% precision.

This research proposes a DL-based framework for predicting the short-term SA of autistic students. First, a univariate LSTM forecasting model is proposed to provide early predictions of the students' SA behavior dependent upon their attendance history. Expanding upon this, a multivariate LSTM model is then employed by enriching the data source with the students' maladaptive behavior history (e.g., aggressive behavior). The maladaptive behavior data is collected every day the student attends school. As shown in Fig. 3, adding maladaptive behavior improves prediction accuracy and precision while it slightly decreases prediction recall. These results encourage us to dig deeper to investigate the relationship between maladaptive and SA of autistic students. Such investigation will help design more customized SA interventions that consider these two essential phenotypes of ASD. For example, more customized in-class learning activities or interventions could be implemented to improve the students' adaptive behavior, which possibly could result in better school attendance.

From a practical perspective, it is of value to know for how far ahead the proposed model can satisfactorily predict SA. So, the robustness of the proposed framework is tested against ten different values of forecasting horizon (lead) shown in Fig. 3. For each value, the forecasting performance is evaluated using three different metrics, as shown in Fig. 3, where ten school days is recommended as the maximum forecasting horizon with acceptable accuracy and precision of (80%). As expected, the overall quality of the prediction decreases as the forecasting lead value increase. It implies that the SA of autistic students might change over time. So, consistent updating mechanisms (e.g., mobile apps) should be in place to record, track, and update attendance. Table 1 shows the superiority of LSTM over other common ML algorithms. Also, the model parameters used across all the experiments are summarized in Table 4.

**Table 1:** SA prediction performance for 10 days lead scenario.

Classifier	Accuracy	Precision	Recall
LSTM	0.80	0.80	0.93
MLP	0.78	0.75	0.84
SVM	0.76	0.75	0.81
RF	0.70	0.72	0.78
KNN	0.55	0.68	0.77
LR	0.51	0.62	0.75

### Long-term SA prediction (scenario I + scenario II)

**Figure 4:**
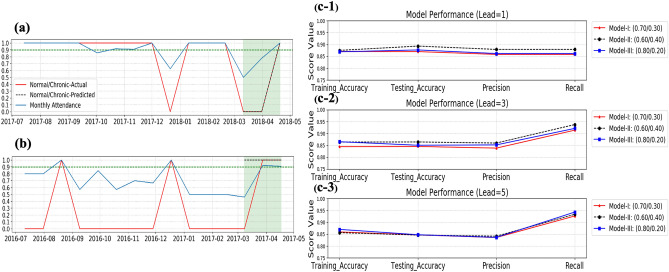
Long-term CA model performance. (**a**) For a student with 100% prediction accuracy (**b**) Another student with 67% prediction accuracy. (**c-1**) Prediction accuracy for a lead value of one day. (**c-2**) 3 days lead value. (**c-3**) **5** days lead value. The best prediction performance achieved by (lead value$=1$) and (0.70/0.30) train/test validation threshold.

MLP and RF algorithms are trained using the CA history of 120 autistic students to predict whether each student will be chronically absent over the upcoming three months. For that, we first tested the robustness of MLP and RF to the data availability represented by the length of the student's enrollment history (lag). In this regard, we considered two scenarios of enrollment history which are twelve and three months. According to Table 2, the MLP algorithm shows better performance in both scenarios. Thus, we conducted further experiments to examine the sensitivity of the MLP algorithm to different settings of prediction horizon length (lead) and train/test splitting threshold, as shown in Table 2. Figure 4 (c1–c3) illustrates the MLP sensitivity to the experimentation settings where it shows the best prediction performance occurs at (lead value $=1$) and (0.70/0.30) train/test validation threshold. MLP outperforms ML algorithms, as Table 2 shows. Model parameters used in the experiments are summarized in Table 4.

**Table 2:** CA prediction performance for 12- and 3-months lag scenarios.

Classifier	Training accuracy	Testing accuracy	Precision	Recall
**Scenario (I): 12 months lag**				
MLP (0.70/0.30)	1.0	0.91	0.94	0.94
RF (0.80/0.20)	0.96	0.85	0.91	0.90
SVM (0.80/0.20)	0.94	0.87	0.87	0.88
**Scenario (II): 3 months lag**				
MLP (0.70/0.30)	0.91	0.81	0.87	0.84
RF (0.70/0.30)	0.93	0.79	0.87	0.90
SVM	0.92	0.86	0.85	0.87
**MLP validation settings**				
Lead (days)	1	3	5	
Train/test split	(0.80/0.20)	(0.70/0.30)	(0.60/0.40)

Our results also highlight the possible relationship between maladaptive behavior and SA of autistic students. More research effort is needed to address this issue quantitatively through different techniques, such as social networks and association mining algorithms. In our opinion, the more the dynamics of ASD phenotypes are investigated, the more the SA interventions will be customized and efficient. Moreover, these research results are expected to encourage school districts to collect, track, and intelligently analyze school-related data, which will result in the improvement of overall education quality.

## Conclusion

Ideally, there would be a simple formula expressing the risk factors for autistic students for school absenteeism. However, the heterogeneity of the population is reflected in our results that one cannot state risk for a group, but rather with sufficiently sophisticated analyses, prediction can be made for individuals. Motivated by the capability of DL algorithms to learn complex patterns, this research contributes to the SA literature by proposing a framework for predicting short-and long-term SA for autistic students. This contribution might assist school districts and caregivers in predicting SA on a daily basis, which is supposed to add to the benefits of predicting those at risk of SA based on other factors identified in the literature. School districts are expected to depend on SA prediction to intervene effectively through (1) timely allocating their attention and resources to specific students and (2) tailoring their school-based activities according to the expected SA behavior of the students. We suggest using our work as a complementary step after diagnosing students at high risk of SA. This is supposed to help practitioners plan interventions to ameliorate SA earlier and with increased effectiveness.

Methodology-wise, ML- and DL-based frameworks are proposed for the SA and CA prediction of autistic students. First, the input data is modeled as a time series to represent the students’ attendance and maladaptive behavior history. LSTM algorithm is used for short-term SA prediction. Moreover, MLP and RF algorithms are then used for long-term CA prediction. Both models show a promising capability to predict SA and CA behavior for ten school days and three months ahead, respectively. The results are expected to help in designing customized interventions to manage SA effectively. Future research includes (1) improving the adopted algorithms' performance through hyperparameters optimization and (2) enriching the proposed framework's data source using other characteristics and behaviors to predict SA and CA.

## Methods

This research introduces an ML- and DL-based framework to handle short-term SA and long-term CA problems for autistic students. LSTM algorithm is used for the first problem. In this regard, univariate and multivariate forecasting models are built. Students' attendance history is used as input for the univariate model, while the multivariate model considers the history of students' maladaptive behavior as another data input. The univariate model predicts students’ SA based on their attendance. In contrast, the multivariate model depends on students’ attendance and maladaptive behaviors to predict their SA. For the CA prediction problem, the individual characteristics are added to the attendance history to enrich the data source. Two different scenarios are also hypothesized for students with long and short attendance history, as detailed later.

### Data description

This research targets a population includes 120 autistic students who have an average age of six years, and of which 79% are male, while 21% are female. The sample has an attendance rate of 90%, while 23% are reported chronically absent. The participants show different individual characteristics in term of the types of medication, diet restrictions, and allergies. The data is collected from the Institute for Child Development (ICD) in the area of Binghamton, NY, where the informants are either the parents or legal guardians. The ICD is a private special education school that primarily provides services to autistic children or children with developmental disabilities. Table 3 provides more details, including a demography survey of the targeted population. The data has 50 k instances representing the individual history of 120 students over their enrollment duration in the ICD. The data covers 14 features related to the students’ demographic and individual characteristics in addition to their attendance history. Demographic features include students' age and gender, and the features related to the individual characteristics include the type of diet restrictions, allergies, medication, diagnosis, and six different standard skills such as motor, social, and living skills. The attendance history is represented by the type and reason of absence as excused or non-excused, in addition to a daily SA status showing whether students miss or attend the school. The research presented in this study was approved by the Binghamton University’s Institutional Review Board (IRB). Also, all methods utilized in this study for data collection were carried out in accordance with relevant regulations. The informed consent was waived off in this study and it was approved by the Binghamton University Human Subjects Research Review Committee (HSRRC), which is the IRB responsible for the review of research.

**Table 3:** Demographic characteristics of the targeted population.

Gender	Male	Female
	0.79	0.21

Age	$\le 5$	> 5
	0.65	0.35

Medical restrictions	With	Without
	0.45	0.55

Diet restrictions	With	Without
	0.58	0.42

Allergy restrictions	With	Without
	0.55	0.45

We first investigated whether the students’ individual characteristics (e.g., communication skills, motor skills, emotional control, and others) are significant predictors of their SA behavior. This investigation is motivated by the lack of research that addresses the relationship between individual characteristics and SA^7^. Statistical hypothesis testing is applied, and the results depicted in Fig. 1 show no association between these characteristics and the SA of the targeted population. The results also support our hypothesis that (1) SA is heterogeneous and should be predicted at the individual level, (2) SA is better predicted depending on its history. The association between maladaptive behavior and SA is discussed in the literature^7^. Therefore, maladaptive behavior will also be used, in this research, to predict SA. This is also supposed to help design customized interventions to possibly improve SA behavior that considers different ASD phenotypes. In this research, we mainly use the students’ attendance history to predict their future attendance patterns. Therefore, the past SA patterns are used as features, while the labels are the future SA patterns. Features and labels are both binaries where 1 and 0 refer to attendance and absence events, respectively. For example, a feature vector of (110) elements means that the student only missed the last day of the past 3 days. Similarly, a label vector of (111) elements means that the student will not miss any of the upcoming 3 days.

### Short-term SA prediction

#### Data preprocessing for short-term SA prediction

To predict short-term SA, the history of students' attendance and maladaptive behavior is first modeled as a time series. Data transformation includes binary encoding of attendance time series (1: attendance, 0: absence) and normalizing the time series of maladaptive behavior. Then, the data is restructured to take the shape of supervised ML-like data using a rolling forecasting technique such that a sequence of $\left(i-l\right)$ past events are used to predict the future event $\left({A}_{i}\right)$ at time $\left({T}_{i}\right)$ where $\left(l\right)$ is the value of the lag parameter. Thus, the entire time series of each student is partitioned into given labels of $\left(N\right)$ binary sequences each of length $\left(l\right)$ as features in addition to events $\left({A}_{i}\right)$, to be predicted. For validation purposes, the data is split using three training–testing thresholds, as will be illustrated later. Other secondary data cleaning steps are also accomplished.

#### LSTM algorithm

LSTM is a popular recurrent DL algorithm that is used to mine the hidden patterns of sequential data^34^. Many LSTM variations have been introduced to enhance its capability (e.g., diamond LSTM and bidirectional LSTM)^34^. The LSTM areas of application are manifold, which include time series analysis, natural language processing, and others^34^. In this research, LSTM will be used for the first time to predict the SA behavior among autistic students.

In this research, the SA of each student is modeled as a time series. Unlike the typical forecasting techniques (e.g., ARIMA and SARIMA), LSTM is known for its capability to learn the long-term dependencies of sequential and temporal data^34^. For this reason, LSTM will be used in this research for short-term SA modeling and prediction. It is worth mentioning that typical forecasting techniques (e.g., SARIMA) perform well on the seasonal and linear time series. However, they are less powerful to capture the long-term dependencies of sequential data than DL (e.g., LSTM)^34^.

Opposite to the typical DL algorithms, the neurons at each hidden layer are replaced by memory cells that work together with three types of gates: input, forget, and output gates. This characteristic enables the LSTM algorithm to avoid the gradient vanishing problem. In this sense, LSTM is proven in the literature for its superiority of learning and predicting long sequential data^34^.

**Figure 5:**
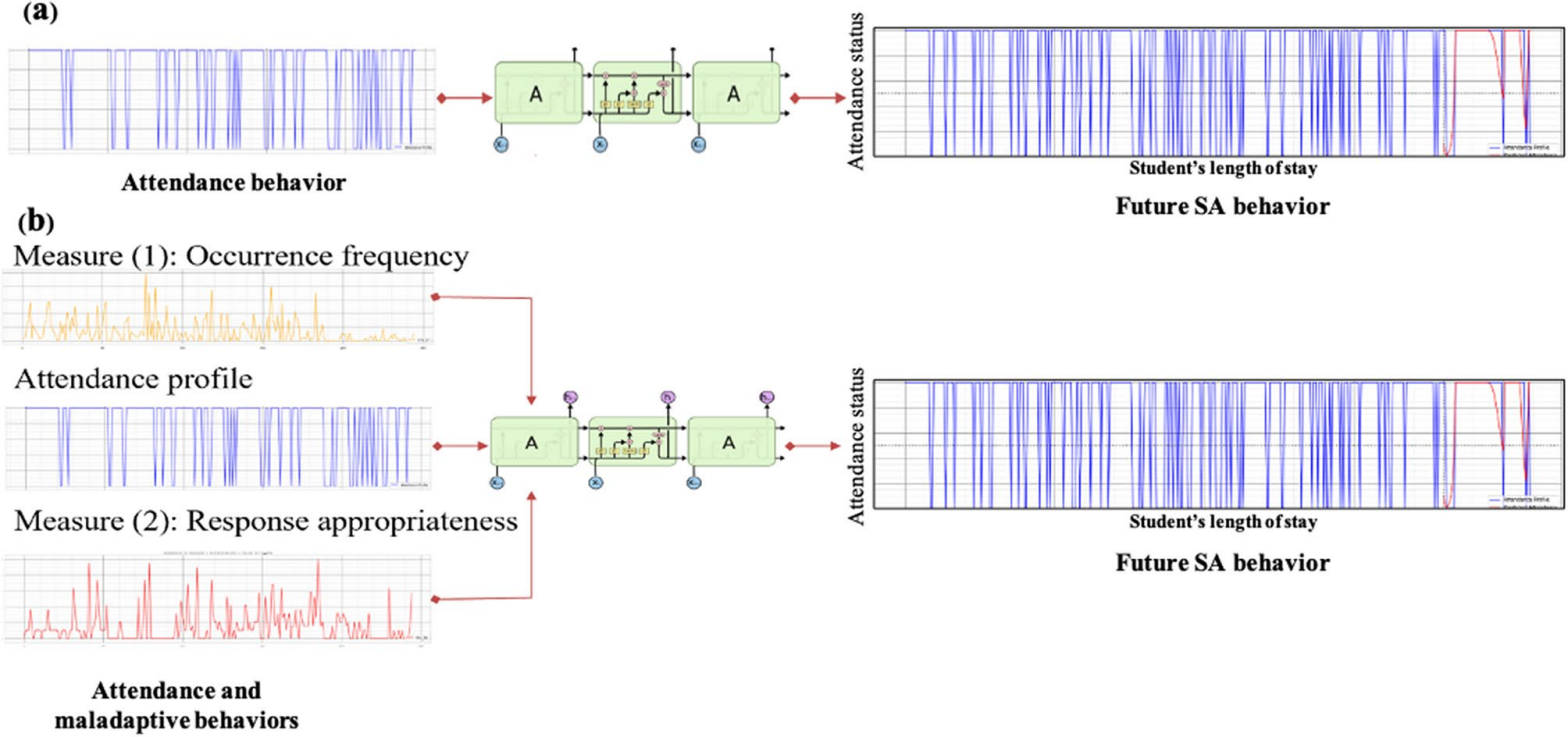
Schematic diagram of the proposed framework (**a**) univariate and (**b**) multivariate models.

To fulfill the scope of this research, univariate and multivariate LSTM forecasting models are built. The time series of students' attendance history are used to train the univariate model as a single input. However, the dataset of the multivariate model is enriched by adding the time series of students’ maladaptive behavior in addition to school attendance. Figure 5 illustrates how the proposed model works.

LSTM algorithm with a rolling forecasting technique is employed in this research to predict future SA. Similar to any DL algorithm, LSTM performance is a function of multiple architectural parameters (a.k.a hyperparameters). Tuning these parameters is critical to optimize LSTM accuracy. Multiple optimization algorithms have been introduced in the literature for this purpose^34^. Parameter’s optimality is beyond our scope in this research because the main focus will be on the introduction of a new framework for SA prediction for autistic students.

SA prediction is addressed as a forecasting problem in this research. Therefore, LSTM performance is also a function of two main forecasting parameters: lag and lead. While lag refers to the amount of history needed to predict the next future event, the lead parameter's value represents the number of future events that could be predicted at once using the given lag value. Table 4 summarizes all the LSTM hyper-parameters values, which include the forecasting lag/lead values, adopted in this research.

**Table 4:** LSTM and MLP hyper-parameters and forecasting values.

Parameters	LSTM algorithm	MLP algorithm
Number of hidden layers	3	3
Activation function	“Linear”	“tanh”
Batch size	32	20
Number of epochs	100	100
Optimizer	“Adam”	“lbfgs”
Lag (days)	5	–
Lead (days)	1	–

Three training–testing split settings are employed for better model validation. Each of these settings is embedded with a rolling forecasting technique that trains the LSTM model using different data portions. In the same regard, accuracy, precision, and recall are adopted to evaluate the model's performance for each of the validation settings. Accuracy reflects the model's overall prediction quality, while the two other metrics check the model's capability to predict the attendance events correctly. Figure 4 shows the model performance over different validation settings.

### Long-term CA prediction

#### Data preprocessing

In long-term CA prediction, the main objective is to predict whether a particular student will be chronically absent over the upcoming three months. This problem is handled as a pattern recognition problem using MLP and RF algorithms. A combination of a 12-month attendance history and 14 individual characteristics (e.g., medical restrictions, allergy restrictions, and atypicality score) have been used as features. Binary encoding is used to model the monthly attendance history as a binary sequence in addition to the individual binary characteristics (e.g., medication and allergy restrictions). Moreover, the individual numerical features (e.g., age) are normalized. The future CA status is labeled as a binary sequential pattern. For example, (100) means the student will be chronically absent in the second and third months.

Data balancing is necessary to avoid learning bias. Therefore, the input data is also balanced using the standard oversampling technique. Different training–testing splitting thresholds are applied to validate the model. This step will be discussed in detail later in this section. To further validate its robustness, we applied our model to a hypothesized scenario where some students have a short history of school enrollment (three months). The results show our framework's ability to predict CA even for recently enrolled students with a relatively short CA history.

#### MLP and RF algorithms

In this research, long-term CA behavior is also predicted. The problem is formulated as a pattern recognition problem. Each pattern represents the status of students' CA for three months ahead. MLP and RF are two commonly used algorithms for pattern classification problems in the literature^35,36^.

MLP is one of the most common ANN with a broad spectrum of applications. It has a powerful capability to approximate non-linear functions by learning the hidden complex patterns in large, complex, and noisy data^35^. MLP architecture consists of one input and one output layer in addition to at least one hidden layer. Inspired by the human brain structure, each layer includes multiple neurons that work as knowledge processing units. Neurons in each layer are connected to the other layers' neurons through artificial links that hold some value of weights. The backpropagation algorithm is commonly used to train MLP and optimize its weights such that the error function converges to its global or local minima.

RF is a state-of-the-art machine learning algorithm with outstanding prediction and feature selection performance^36^. RF works simply as an ensemble learning algorithm that aggregates $\left(N\right)$ independent and deep tree predictors into one powerful final model. In this sense, RF has an outstanding capability to learn complicated and irregular patterns^36^. In more detail, the FR algorithm trains $\left(N\right)$ independent trees $\left({f}_{b}\right)$ using different portions of the training data $\left\{\left({X}_{b},{Y}_{b}\right)\in \left(X,Y\right)\right\}$. Then, the final model $\left(F\right)$ is made by averaging the performance of all the individual models $\left({f}_{b}\right)$.

MLP and RF have been used to handle the long-term CA prediction as a pattern recognition problem. We applied both algorithms considering two scenarios of twelve- and three-month long histories of school attendance. These scenarios are hypothesized to investigate the robustness of the proposed framework to predict CA for students with different attendance history lengths. The hyperparameters optimization step is not considered as it is beyond the scope of this research. Table 4 summarizes the model parameters that are used for each algorithm.

To validate the adopted models' performance, we tested the results using different data splits to train the models using different data portions. In addition, accuracy, recall, and precision metrics are also used to investigate the quality of our predictions.
